# Nursing experience and leadership skills among staff nurses and intern nursing students in Saudi Arabia: a mixed methods study

**DOI:** 10.1186/s12912-024-01750-1

**Published:** 2024-02-02

**Authors:** Bayan Alilyyani, Emad Althobaiti, Muath Al-Talhi, Talal Almalki, Tariq Alharthy, Mohammed Alnefaie, Husam Talbi, Ahmed Abuzaid

**Affiliations:** Department of Nursing Management and Education, College of Nursing, Taif University, 2425 Taif, Saudi Arabia

**Keywords:** Leadership, Skills, Experience, Nurses, Intern, Student, Quantitative, Qualitative

## Abstract

**Background:**

Nurse leaders have a crucial impact in healthcare settings. Hospitals require qualified nurses with leadership skills to provide healthy work environments and enhance the outcomes related to staff nurses and patients. This study aimed to investigate the effect of nursing experience on leadership skills among staff nurses and intern nursing students.

**Methods:**

A mixed methods design was applied (quantitative survey design for quantitative part and open-ended questions for qualitative part). Convenience sampling of staff nurses and intern nursing students in Saudi Arabia was applied. There were148 participants who completed the survey of the quantitative part, and 50 of them completed the qualitative part. Participants completed the Leadership Practice Inventory Questionnaire. SPSS v26 was used to analyze quantitative part, and thematic analysis was used to analyze qualitative part.

**Results:**

This study found a significance difference among participating groups regarding to the years of experience (*F* = 5.05, *p* = 0.00). Three themes were found for the qualitative part which were strategies to enhance leadership skills, factors affecting leadership skills, and obstacles facing participants related to leadership skills. The qualitative data also revealed that participants found that clinical supervision and education ways to enhance the leadership skills, while work pressure, work environment and communication were obstacles of developing their leadership skills.

**Conclusion:**

Leadership skills are considered as a significant component of the function of qualified nurses and should be viewed as central to intern nursing students’ learning development. Moreover, leadership skills are essential for the patient and organization outcomes. Nursing colleges and educators play an important role in enhancing to leadership skills as well as experience. Nurse leaders can create healthy care environments that have high quality and safety for patients. Management systems in healthcare organizations must motivate and support clinical leaders who can recognize both individual and clinical requirements and address current issues in their field.

**Supplementary Information:**

The online version contains supplementary material available at 10.1186/s12912-024-01750-1.

## Background

Healthcare organizations need leaders who can successfully overcome challenges, meet them head-on, and carry on their organization’s mission and vision, and courageous priority [1, 2]. This means that leaders need to make decisions which influence them to have the ability to deal with any new problems [1]. The objective of raising the number of qualified nurse leaders is not a short-term one, but it is a long-term strategic approach which makes leadership capacity in multi-professional healthcare organizations a difficult process [3]. Healthcare organizations have the responsibility to provide suitable support structures and resources to support nurse leaders, especially newly graduated nurses [3].

High levels of patient satisfaction and improved outcomes are carried out by effective leadership [4]. In addition, delivering high-quality patient care, clinical safety, and staff retention all depend on effective leadership, which is crucial for healthcare sectors [5]. Previous studies have highlighted the effect of nurse leadership on all nurses, patients, and organizations outcomes [6, 7]. For example, the results of a systematic review conducted by Wong et al. [7] showed that there are relationships between leadership and patient outcomes such as patient satisfaction, patient mortality, and patient safety.

Developing leadership skills is one of the important elements that each nurse should focus on. Newly graduated nurses, more specifically, should develop their leadership skills. After creating Vision 2030, a program that contributes to a number of developments in the domains of health delivery systems, nursing, trade, education, communications, science, and technology, Saudi Arabia has entered a new era of progress and wealth [8]. Enhancing the health delivery system to improve population health status is one of the objectives of Saudi Vision 2030 [8]. The Vision also strives to highlight nursing and its crucial position in the multidisciplinary healthcare team, as well as to make nursing and medical support personnel more appealing as a preferred career path [9]. The Saudi Vision 2030 encourages nursing leaders, educators, and practitioners to coordinate their initiatives to transform nursing [8]. To promote the nursing profession and enhance overall health care in the Kingdom of Saudi Arabia, nursing leaders and educators, decision-makers, and researchers must collaborate and adhere to the 2030 Saudi Vision [9].

No matter their position or level of experience, nurses are expected to play a leadership role [10]. The role of nurse leaders is crucial to newly graduated nurses because they not just offer resources and recognition, but also they promote positive working environments with access to support and communication [11]. Regardless to any interventions, clinical leadership skills for newly graduated nurses considerably enhance over the course of the first year of practice [12].

This study may benefit nurse leaders in the healthcare system to enhance their leadership skills which in turn helps to reach the highest quality of services. It also could motivate nurse leaders to share their leadership experience with staff nurses and involve them in leadership tasks. This helps to enhance the work environment and outcomes related to patients, nurses, and organizations. Also, it could help intern students to explore and develop their leadership skills. Thus, the goal of this study was to examine the effect of years of experience on leadership skills among staff nurses and intern students. Also, this study aimed to identify leadership skills and methods of developing them for staff nurses and intern students.

## Materials and methods

### Study design

This study used a mixed methods design. More specifically, embedded design was applied to collect analyze both quantitative and qualitative data simultaneously. The focus was on quantitative data and used qualitative data to deep understand the phenomenon. Using mixed methods allowed to get a comprehensive view of the of years of experience and leadership skills and to identify leadership skills and methods of developing them among staff nurses and intern students.

### Setting

This study was conducted in the Western region of Saudi Arabia. Thus, all staff nurses and intern nursing students who met the inclusion criteria were invited to participate in the study.

### Sample

This study used non-randomly convenience sampling. By calculating an estimated the population which was 10,000 nurses and 700 intern nursing students in the Western region [13, 14], a confidence level of 95%, and a margin of error of 5, the sample size was determined to be 370 nurses and 249 intern nursing students.

The Inclusion criteria were registered nurses and intern nursing students including (both genders and all ages) who worked in Saudi hospitals in Western region of Saudi Arabia, were able to complete the surveys and qualitative questions in English, and agreed to participate in the study. However, the exclusion criteria were leaders or managers, interns in other healthcare professions, individuals who refused to participate in the study, and nurses and intern students who couldn’t complete the surveys and qualitative questions in English.

### Instruments

For the quantitative part: Leadership Practice Inventory-Self Assessment, is a validated questionnaire developed by Posner and Kouzes [15], was used to measure clinical leadership. It consists of 30 items rated on a five-point Likert scale from 1 (Never) to 5 (Always). Internal reliability and factor analysis were measured in the previous study and were acceptable [15]. Cronbach alpha was 0.95, and eigenvalues was greater than or equal to 1.0 and accounted for 59.9% of the variance [15].

Demographics were also collected including, age, gender, nationality, level of education, region, occupation, years of experience, and department.

For the qualitative part: open ended questions were applied. The focus of open ended questions was on ways to enhance the leadership skills of staff nurses and intern students, obstacles facing staff nurses and intern students to enhance their leadership skills, and the effect of education and/or experience on enhancing the leadership skills. Nine questions were developed for qualitative part. The questions were constructed based on the survey used in the quantitative part and previous studied, so participants were able elaborate and explain in depth on their experience (see supplementary file).

### Data collection

Data were collected from staff nurses and intern students in Western region of Saudi Arabia. Quantitative data were collected through a survey using Google Forms. Before sending the survey out to the participants, the researchers checked the link to ensure the validity of the link, and the survey was clear and organized. The collecting data were then transferred to the Excel sheet for coding and organization. After verification, data were transferred to SPSS to perform the analytical tests. Qualitative data were collected through using open-ended questions filled by participants after completing the surveys.

### Data analysis

For the quantitative part: data analysis was performed using SPSS v26. Descriptive statistics was used to measure the demographic data of the participants. Means and standard deviations were measured. The differences between staff nurses and intern students were analyzed by using unpaired t-tests. The level of significance was *p* < 0.05. The reliability of Leadership Practice Inventory-Self Assessment scale was measured by using Cronbach’s alpha.

For the qualitative part, thematic analysis was applied. The results of qualitative part were organized into different themes based on participants’ responses by using content analysis [16]. After collecting data of the qualitative part, data were organized and coded to ensure the accuracy of the analysis. Then, data were analyzed by using content analysis and organized in themes based on the participants’ responses. Finally, data were reported based on insights gotten from the analysis.

### Ethical consideration

Ethical approval was obtained from Taif University (IRB NO: 44–082; Date: 09-11-2022). No study-related actions had begun before receiving IRB approval. Participants were asked about their agreement before they started competing the surveys and open questions. Completing the surveys was considered a volunteering agreement. Neither staff names or any private social details were collected. The participants had the right not to participate in or withdraw from the study at any stage.

## Results

### Quantitative results

#### Participants’ characteristics

The demographics of the participants are illustrated in Table 1. A total of 148 participants completed the survey. As shown in Table 1, the majority of the participants were female 59% (*n* = 87), and 64% (*n* = 95) of them aged between 23 and 30 years. Most of the participants 76% (*n* = 112) were Saudi, and around 88% (*n* = 130) of them held a bachelor’s degree in nursing. 47% (*n* = 69) of them lived in Taif city. A large part was registered nurse 64% (*n* = 94). Most of them had less than a year of experience 46% (*n* = 68). The plurality of the participants worked in oncology department 25% (*n* = 37) and other departments 29% (*n* = 43).

**Table 1:** Participants’ characteristics

Age	Frequency	Percentage
23–30	95	64.2
31–35	25	16.9
36–40	21	14.2
Above 40	7	4.7
Total	148	100
**Gender**		
Male	61	41.2
Female	87	58.8
Total	148	100
**Nationality**		
Saudi	112	75.7
Non-Saudi	36	24.3
Total	148	100
**Level of Education**		
Diploma degree	8	5.4
Bachelor’s degree	130	87.8
Master’s degree	10	6.8
Doctoral degree	0	0
Total	148	100
**City**		
Makkah	52	35.1
Jeddah	27	18.2
Taif	69	46.6
Total	148	100
**Occupation**		
Intern Nursing Student	54	36.5
Registered Nurse	94	63.5
Total	148	100
**Years of Experience**		
Less than 1 year	68	45.9
Between 1–3 years	23	15.5
More than 3 years	57	38.5
Total	148	100
**Department**		
ED	23	15.5
ICU	15	10.1
Surgical	13	8.8
Medical	11	7.4
Oncology	37	25.0
Cardiac	6	4.1
Other	43	29.1
Total	148	100

#### Relationships between leadership practice and demographics

An independent sample t-test and ANOVA were used to analyze the relationship leadership practice and demographics. As shown in Table 2, there is significant relationship between leadership practice and both nationality (*t* = -2.75, *p* = 0.00) and occupation (*t* = 2.42, *p* = 0.01).

**Table 2:** Relationships between leadership practice and demographics

	Age	Gender	Nationality	Education level	City	Occupation	Department
**Leadership Practices**	*F* = 2.64	t = -1.63	*t* = -2.75	*F* = 1.06	*F* = 0.88	*t* = 2.42	F = 1.12
	*p* = 0.05	*p* = 0.10	*p* = 0.00	*p* = 0.34	*p* = 0.41	*p* = 0.01	*p* = 0.34

Note: *p* < 0.05

#### Reliability of the leadership scale

Table 3 shows the reliability of the scale using Cronbach’s Alpha. The results revealed that the Leadership Practices Questionnaire with thirty items (α = 0.97) was found reliable and acceptable.

**Table 3:** Reliability of the leadership scale

No of Items	Mean	Std. Deviation	Cronbach’s Alpha
30	3.59	0.89	0.97

#### Differences in means between two groups (intern nursing students and registered nurse)

**Fig. 1:**
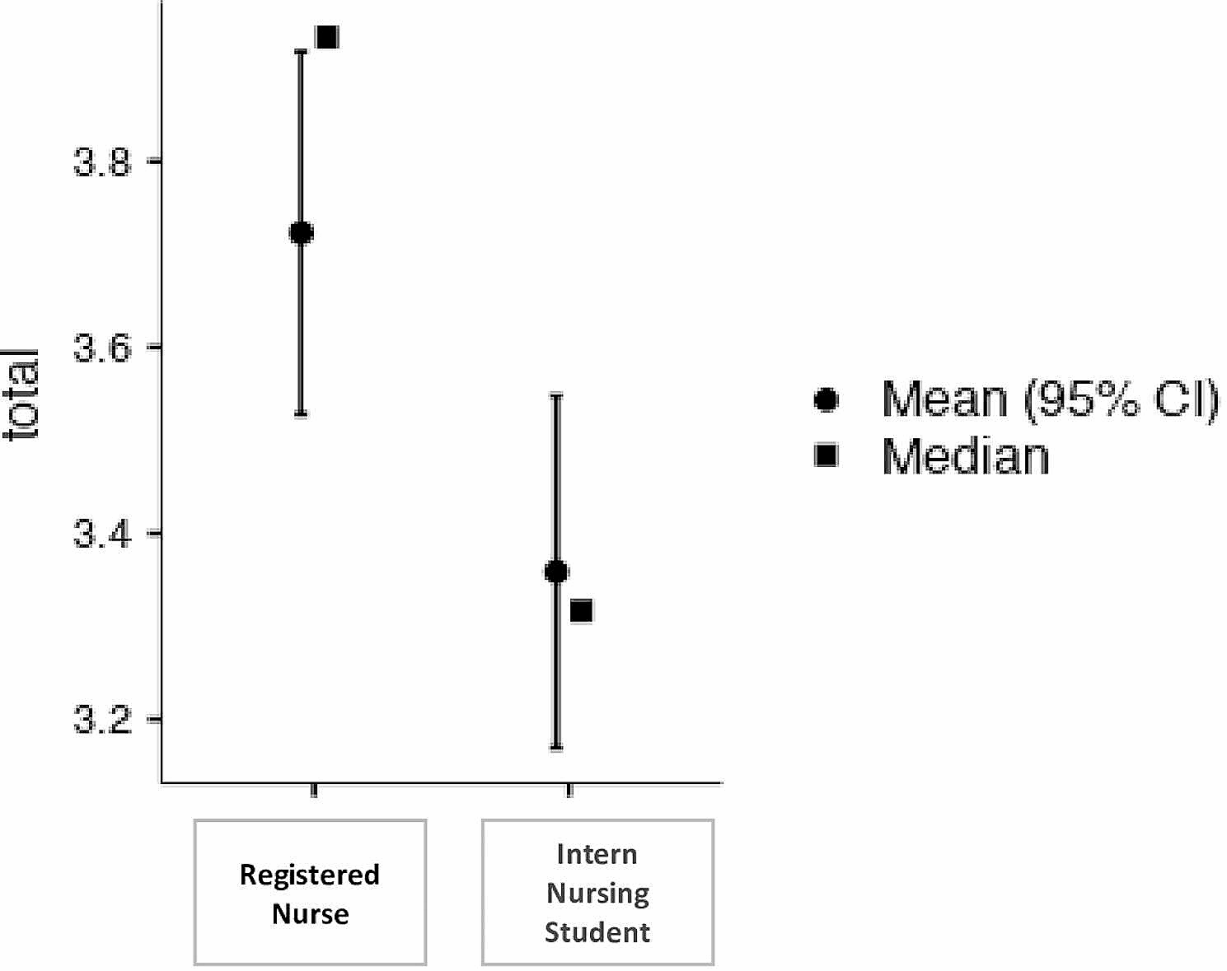
Differences in means between two groups

An independent sample t-test was conducted to compare Intern Nursing Students and Registered Nurses. As shown in Table 4; Fig. 1, there is a statistical difference between two groups (Intern Nursing Student and Registered Nurse) regarding to leadership practices.

**Table 4:** Differences in means between two groups (intern nursing students and registered nurse)

No of Group	N	Mean	Median	Std. Deviation	Std. Error	t	df	***p***
Group 1	54	3.36	3.32	0.71	0.096	2.42	146	0.01
Group 2	94	3.72	3.93	0.96	0.099			

Note: Group 1 (Intern Nursing Students), Group 2 (Registered Nurse)

*p* < 0.05

#### Differences in leadership practice regarding to years of experience 

Table 5 illustrates the differences in leadership practice regarding to the years of experience. There is a significant difference among participating groups regarding to the years of experience (*F* = 5.05, *p* = 0.00).

**Table 5:** Differences in leadership practice regarding to years of experience

	Sum of Squares	df	Mean Square	F	***p***
Years of experience	7.66	2	3.83	5.05	0.00
Residuals	110.06	145	0.75		

Note: *p* < 0.05

#### Qualitative results

This part of the results is related to qualitative questions that were provided for the participants. The number of participants completed the qualitative questions were 50 nurses and intern students. The open-ended questions were sent to all participants who completed the surveys, and 50 of them were able to complete the qualitative part. By reaching the participant number 45, we reached the data saturation. Participants were asked questions regarding to strategies to enhance their leaderships skills, factors affecting their leadership skills, and obstacles facing them related to leadership skills. The following three themes were categorized based on the participants responses.

#### Strategies to enhance leadership skills

The participants mentioned that developing of education curriculum, training, workshops, reviewing the research regarding to leadership, applying the varies styles of leaders, and being in emergency situations make them to be decisive and precise to promote the skills of leadership. Moreover, demonstrating principles such as confidence, honest, respect, guidance, and trust could help to develop their leadership skills. Furthermore, open communication, active listening, and providing some advice from experienced leaders play a crucial role in developing leadership skills of intern nursing students and staff nurses. Most of the participants believe that skills shall be applied rather that only taught as theory; being involved in decision making and letting them to decide and share their opinions, it’s considered as an opportunity to lead. Other side, the supervisor must take a round to look and identify their needs. Finally, nurses must be initiative to enhance their leadership skills.

#### Factors affecting leadership skills

Many factors can improve the impact of the leadership skills of nurses and intern students in the work environments, but the most influencing in developing leadership skills are education and experience. Education plays an important role, and it is the cornerstone that enables leaders to make decisions and helps solving problems through specific strategies based on reliable scientific studies and research. Education helps to learn different leadership styles that support them to lead staff effectively.

Experience is the most effective way to enhance leadership skills, and it is the greatest teacher. The more you have experience, the more you know how to deal with different situations. Also, nurses who have more experience have a preference over those without experience. Experience is gained through the work environment and from leaders and seniors colleagues. Finally, experience and education are both important for effective leadership, and we cannot rely on one without the other because they complement each other.

#### Obstacles facing participants related to leadership skills

Nursing staff and intern nursing students face many obstacles. However, after analyzing the answers of both nursing students and nurses, there were major reasons: work pressure, work environment, communication, experience, and self-confidence.

Work pressure is the most common obstacle affecting nurses and nursing students. The main causes of work pressure are shortages of nursing staff, non-cooperative staff with students, long 12-hour shifts, and multitasking. The work pressure affects students’ ability to learn from the senior nurses and develop their skills. Based on the answers of the research participants, it is too many patients for one nurse, so there is no time to learn. Nurses are so busy teaching the students and have no time to continue education. This brings us to the second problem, which is experience.

Experience is an important reason because it gives intern nursing students the confidence to participate in the medical care process, express their ideas and opinions, and develop the department. With no experience, the students lose their confidence, so they avoid getting close to other nurses interacting in the work environment.

The work environment is another obstacle because many things are related to it such as health facility capabilities, management, facility policy, managers, and co-workers. Examples of this include negligence, interpersonal conflicts, nurses not being involved in decision-making, no appreciation for work, and dominant roles of the boss on a subordinate in the workplace.

Communication is one of the most important obstacles if it is not the most important factor for improving the relationship between co-workers and raising the quality of work. Several factors affect the quality and effectiveness of communication, such as language, work pressure, and old management styles that depend on following orders without listening to the other party.

There are many other obstacles such as, time management, income that does not match the effort, lack of opportunities, stereotypical view of nurses, life problems and loss of passion. Table 6 provides some participants quotes related to each theme mentioned above.

**Table 6:** Participant quotes

Theme	Participant quotes	Occupation
1-Strategies to enhance leadership skills	“Be confident and voice your concerns and opinions” OR “Enhance the way of managing time and distribution of work”	Intern Nursing Student
	“Learn the basic skills from leader they have more experience”	Intern Nursing Student
	“Let them to do more practice so it can build confidence”	Registered Nurse
	“Independence, everyone should be independent, at least once in a leadership role to enhance their hidden skills”	Registered Nurse
2-Factors affecting leadership skills	“Education can affect it but not seriously, if there is true collaboration”	Intern Nursing Student
	“Yes, leadership skills are acquired and can be obtained by learning”	Intern Nursing Student
	“Of course, the more you had experience, the more you know how to deal with different situation”	Registered Nurse
	“Yes, experienced individuals have advantage from those who does not have”	Registered Nurse
3-Obstacles facing participants related to leadership skills	“The senior nursing staff in the hospitals, because they follow the old style of nursing practice “obedience to doctor order, don’t contribute in the health management of the patient or choices of health care, JUST DO WHAT THE DOCTOR SAY!”	Intern Nursing Student
	“The existence of some environments that don’t encourage leadership abilities, and their preference for the dominant roles of the boss on a subordinate in the workplace”	Intern Nursing Student
	“No clinical instructor that guides them in the area. Staff and head nurses cannot accommodate students in the field since there is shortage and with time constraint. In our country, clinical instructors are with the students during their clinical exposure. It is the responsibility of the clinical instructor to teach and guide the students. But what I am seeing in the institution here, the students are left without the supervision of the clinical instructors. They rely on head nurses and staff nurses to teach their students receive the best exposure”	Registered Nurse
	“A lot but most important things mange time socially during the work time on hospital and the home”	Registered Nurse

## Discussion

This study reports the results of the quantitative part of association between nursing experience and leadership skills among staff nurses and intern nursing students in Saudi Arabia. The main findings were that years of experience and occupation were highly significant among intern nursing students and nurses. The findings of this study demonstrate significant differences between nurses and intern nursing student in their leadership skills. Nurses were more likely to have higher level of leadership skills because they relate to expertise when compared to intern nursing students.

These findings are consistent with previous research in the field which found that nurse leaders usually be in leadership positions without having requisite knowledge, and they have to acquire the knowledge form their experience [17]. This emphasizes that leadership skills can be acquired from the experience. Moreover, leadership is considered as an essential aspect in nursing, so it should be the focus of education for undergraduate students [18]. Nursing students have difficulty in understanding leadership because of gaps between nursing education and practice [2].

According to Rosser et al. [19], intern nursing students may have inappropriate learning experience such as poor leadership and negative experience during their practice from their supervisors or preceptors in the clinical settings which affect their leadership skills that can be acquired during internship duration. In addition, this will affect their confidence about the levels of knowledge and tasks that should be learnt [20]. Therefore, students could feel unsure about the expectations from them in the clinical practice [21]. The role of nursing practice is very critical for nursing students as it provides the needed confidence and help them to manage any uncertainties could have about both knowledge and skills [20].

Leadership skills are usually shown as a continuous development through the career path beginning with pre-registration schooling. However, evidence shows that there has been inconsistency within undergraduate nursing education [22]. There is a need for linking theory and practice with the nursing professional values [18]. It might appear nursing students can develop knowledge and think more quickly and intuitively as registered nurses do. Nurses may also have specific experience related to a group of patients where they work while nursing students can only begin to understand the basic nursing care during a clinical placement [20]. Both registered nurses and intern nursing students have different experiences of leadership because they expose to the same guidance, culture, and polices [5].

In addition, the findings of this study did not demonstrate any significant differences in level of education among nurses and intern nursing students. That is in line with a previous study conducted by Handor [23] found that bachelor nursing students and master nursing students can contribute to the leadership qualities in healthcare settings. However, another study found that nurses need to have high level of education and acquire various competencies and skills to enhance their leadership skills through continuous and post graduate education [24].

In Saudi Arabia, the Nursing Internship Program (NIP), is a mandatory program to qualify the nursing students to apply their clinical nursing skills under supervision and acquired a completed four academic years. The Nursing Internship Program is a one-year program implemented in the end of academic years. The NIP is fully paid and organized and monitored by the university, while NIP is provided by the internship facilities [25]. The NIP allows students to face new challenges and put their knowledge and skills to the test [20]. The students have their choices to select the hospital and the training period. Furthermore, it can influence in which nursing specialties a student chooses [25].

Most of the hospitals in Saudi Arabia provide a full orientation to each department such as (medical surgical ward, emergency department, outpatient department, etc.) which leads to self-improvement and work commitment [26]. On other hand, a few hospitals provide an orientation in management department. At the end of the program, students must be a licensed nurse after passing the Saudi Nursing Licenses Exam (SNLE). The NIP allows nurse interns to practice and improve their skills while they are supervised by staff nurses, head nurses, and nurse educators [20]. However, during their internship program, they noticed that staff nurses lacked trust and had high expectations, making the experience challenging and frustrating [27]. They also could experience a lack of trust and high expectations from nurses which could negatively affect their perceptions as intern nursing students [27].

Another problem encountered by the students during their internship program was the lack of clear guidance related to their responsibilities because of the nursing shortage [27]. Nurse are busy with their patients and don’t have time to teach nursing students [27].

The following sections in the discussion focus on discussing the results of qualitative part of this study.

### Clinical supervision

The participants reported that there are many ways to enhance leadership skills in registered nurse and intern student. The most important way was clinical supervision. One of the participants mentioned that clinical supervision is important and said that “Learn the basic skills from leader who have more experience”, and another participant said that “the supervisor must take a round to look and identify their needs”.

Clinical supervision encourages self-learning and improve knowledge, skills and attitudes to empower nurses [28]. Clinical mentoring is a vital process that influences the growth and development of students in various fields, so nursing leaders are needed. Nursing leaders must be role models for students, so students can learn from them the basics of leadership and understand the clinical leadership environment. Assisting and modelling by experienced mentors might help the students to enhance their leadership skills and problem-solving talents through teamwork. It involves having an experienced person who can provide guidance, feedback, and support to the student throughout their learning journey. For example, a clinical mentor can help nursing students practice their clinical skills, assess their progress, and address any challenges they face. Clinical mentoring can also help students develop their leadership skills, as they learn from the mentor’s example and gain confidence in their own abilities. For instance, a clinical mentor can encourage a nursing student to take initiative, communicate effectively, and collaborate with others in a health care team.

Clinical mentoring can have positive effects on the student’s academic performance, professional identity, and career aspirations. The experiences of educators and nurses who influence and mentor students in clinical practice is essential for students learning and improvements [18]. Therefore, clinical supervisors or mentors should determine the nursing student’s learning priorities during the clinical placement. It is also necessary for the clinical supervisor or mentor to understand what motivates students [20]. It is important to have a qualified and competent mentor who can facilitate the student’s learning and development in a clinical setting. Being a role model was an important aspect of clinical nurse leadership. To be a role model, clinical leaders need to reflect on insights and be visible and work in congruence with their personal and professional values. Role models were able to use their skills to mentor others in leading, coordinating and delegating and were proud to work with patients. Role modelling also concerned an ability to develop coping, personal wellbeing and resilience skills [5]. If supervision is insufficient, students could not feel empowered [29]. Therefore, this could negatively impact leadership confidence of newly graduated nurses [5].

### Time management

Time management is about self-management that has an essential impact in achieving the goals of any organizations. There was a participant mentioned that one of the ways to enhance leadership skills is time management and said, “Enhance the way of managing time and distribution of work”. One of the skills that must be acquired by leaders, which makes them effective leaders, is time management because it makes them work smarter and faster and helps achieving goals on time which in turns affects the group to be effective and productive.

The importance of time management makes the group work with greater focus, and organizing time makes leaders to be available for their teams whenever they need them [30]. However, poor time management could lead to delaying work which in turns affects the achievements organizations goals [31]. This also leads to increase pressure and tension among team members which generates some errors and affects patient safety and outcomes [32].

### Education

Some of the participants agreed that the education improves the leadership skills in a way that helps in understating the different leadership styles. Leadership in nursing education gives nurses the required knowledge about the theory of leadership and the skills to implement the theory into clinical practice [20, 33]. Education plays an important aspect in enhancing the leadership skills among intern nursing students. Leadership education can be incredibly valuable for students, as it can help them develop important skills and qualities that can benefit them in a variety of contexts, such as enhances personal development like self-awareness and emotional intelligence which benefit them by understanding their strengths and weaknesses, students can work on improving themselves. Education builds a strong foundation of the leadership concepts, styles, traits, and principals. Overall, earning the leadership theory from the academic curriculum involves a combination of theoretical knowledge and practical application and requires ongoing learning and development. Leadership education provides nurses the appropriate knowledge and skills to become effective leaders [34]. Leadership education is particularly important for staff nurses, as it can help them develop the skills and knowledge needed to effectively lead and manage patient care and it’s important to foster a positive work environment, so it helps staff nurses develop the skills needed to lead and manage teams in a positive and effective way, and prepares for leadership roles such as charge nurse, clinical nurse leader, or nurse manager.

### Experience

Other participants stated that there are factors enhancing leadership skills other than education which is experience. Experience is one of the most important factors to develop leadership skills. Experience could change the understanding of self, and discovers strengths and weaknesses. Thus, experience is one of the most important aspects of nursing because it improves skills because of dealing with a variety of situations while working with patients and teams. Different skills, as a result, are developed such as problem-solving and critical thinking skills, which are essential for providing the best care to patients.

Experience is also important in building effective communication and developing leadership skills [18]. Nurses who have more experience are able to deal with different situations [20]. The ability to comprehend a problem and clarify the factors could be acquired by experience. Experience helps to discover and develop skills that couldn’t cover in the education such as problem solving and managing situations effectively [35]. Experience is said to change one’s understanding of strengths and weaknesses. Student A emphasizes that more experience leads to better ability to deal with different situations. Also experience in nursing improves problem-solving and critical thinking skills, which are essential for providing the best care to patients.

### Work pressure

It is a common obstacle for nurses and nursing students. It is caused by staff shortages, non-cooperative staff, long shifts, multitasking, and incomplete tasks by interns. This affects students’ ability to learn and develop their skills especially leadership skills. Factors that increase work pressure could be work overload, lack of appreciations from leaders [36]. Nurses spend more time on nursing services, and society does not value nurses’ work as much as that of physicians. Thus, these psychological and physical pressures may lead to increase job turnover intentions [37]. Extra workload may affect the focus of nurses and increase conflict among nurses and healthcare providers [38].

### Work environment

It is one of the obstacles mentioned by participants, and it encompasses many factors such as health facility capabilities, management, facility policy, managers, and co-workers. Examples of issues that can arise in the work environment include negligence, interpersonal conflicts, nurses not being involved in decision-making, no appreciation for work, and dominant roles of the boss on a subordinate in the workplace. The findings have highlighted the role of leadership in supporting nurses’ work environment [38, 39].

The healthy work environment that provides adequate resources and has supportive administration help to enhance outcomes related to all nurses, patients and organizations [37]. Healthy work environments reduce negative outcomes such as burnout and bullying and promote positive outcomes such as engagement and [38]. When nurses and nursing students are satisfied with leaders, they feel empowered and involved [29, 40].

### Communication

In nursing, one of the most important factors for improving the relationship between co-workers and raising the quality of work is communication. The future of health care is unpredictable and skilled and effective nurse leaders who have good communication skills are needed to ensure the quality of provided care [41]. However, several factors can affect the quality and effectiveness of communication, such as language, work pressure, and old management methods that depend on following orders without listening to the other party. Leadership styles with high communication skills are associated with higher outcomes such as staff’ empowerment, engagement, and satisfaction [42]. One of the most important problems related to communication in nursing is the old style of used by nurse leaders to guide them in nursing practice, obedience to the doctor’s order, and they do not contribute to the patient’s health management or health care options.

Communication is an acquired skill and expertise needed for successful of leaders and managers [42]. One of the most important factors influencing the style and method of communication between the manager and the nursing staff. Leaders with good communication skills have more engaged and innovative staff whom loyalty to their work is high [42].

## Implications

Nurse leaders establish clear departmental team objectives, increase participating at all levels of an organizational hierarchy, and sustain a commitment to excellence and innovating. They can create a healthy care environment that meets the highest standards for quality, safety, and patient focus [43]. Health Management Systems must create clinical leaders who can recognize both individual and clinical requirements and address current issues in their field. This is necessary because healthcare is becoming more complex [43]. Local organizational cultures are improved by effective leadership to achieve high levels of caring and secure healthcare services. In order to assure the development of the leadership behaviors, methods, and qualities of healthcare professionals, it is essential to recognize that leadership is one of the important determinants of organizational culture [44].

Nursing interns must be aware of the importance of improving their leadership abilities and skills. For example, faculty members should be able to provide a role model for nursing students and assure a secure environment where learning can occur and apply leadership skills in teaching students [45, 46]. Students who acquire leadership from education and theory are able to participate in providing high-quality clinical care and are committed to adhering to their principles in nursing practice [47]. On the other hand, students whose leadership skills are not explored or enhanced can affect them in nursing practice and be dependent on providing care for their patients.

## Recommendations for future studies

More research in this area is required, particularly in Saudi Arabia. Additional studies are needed in this area to implement programs to develop nurses and internet nursing students leadership skills. Also, they measure the effectiveness of programs in order to gain a better understanding of how intern nursing students and registered nurses improve their leadership skills by utilizing a quasi-experiential design, mixed methods, and other valid instruments.

## Limitations

There are some limitations for this study. Because of the small sample size in this study, the results may not be generalizable to all intern nursing students and staff nurse in Saudi Arabia. Furthermore, the convenience sample utilized in this study may not accurately reflect Saudi Arabia’s intern nurse students and registered nurse populations.

## Conclusions

Leadership is one of the essential elements in nursing profession. All nurses should have leadership skills because all of them are leaders. The results of this study emphasize that years of experience plays an important role to enhance leadership skills of both staff nurse and intern nursing students. Nursing education also has an effect on developing leadership skills. Although the concept of leadership in nursing has been improved, there are still some gaps and obstacles facing the profession regarding to leadership that need to be explored and solved.

### Electronic supplementary material

Below is the link to the electronic supplementary material.

Supplementary Material 1

## Data Availability

The datasets generated and/or analysed during the current study are not publicly available due the privacy and confidentiality of participants information but are available from the corresponding author on reasonable request.
