# Supplementary material for: Nursing experience and leadership skills among staff nurses and intern nursing students in Saudi Arabia: a mixed methods study
Source: BMC Nurs. 2024 Feb 2;23:87. doi: 10.1186/s12912-024-01750-1 (PMC10835976; doi:10.1186/s12912-024-01750-1)
Supplement: Supplementary file 1 — Supplementary Material 1 [file 12912_2024_1750_MOESM1_ESM.docx]

**Open ended questions for qualitative part**

1. In your opinion, what are the ways to enhance the leadership skills for staff nurse and intern nursing students?
2. How does your leader motivate you to enhance your leadership skills?
3. How do you stay up to update in your organization?
4. In your opinion, how do education and experience affect enhancing the leadership skills?
5. What do you do to improve your leadership skills?
6. How do you manage your time to improve your leadership skills?
7. In your opinion, what are the obstacles facing staff nurses and intern nursing students?
8. How do you deal with any obstacles that could affect you to enhance leadership skills?
9. Have you been involved in any leadership tasks? Explain your experience?
